# Intolerance of uncertainty heightens negative emotional states and dampens positive emotional states

**DOI:** 10.3389/fpsyt.2023.1147970

**Published:** 2023-03-22

**Authors:** Jayne Morriss, Kimberly Goh, Colette R. Hirsch, Helen F. Dodd

**Affiliations:** ^1^Faculty of Environmental and Life Sciences, School of Psychology, University of Southampton, Southampton, United Kingdom; ^2^School of Psychology and Clinical Language Sciences, University of Reading, Reading, United Kingdom; ^3^King’s College London, Institute of Psychology, Psychiatry and Neuroscience, London, United Kingdom; ^4^South London and Maudsley NHS Foundation Trust, London, United Kingdom; ^5^College of Medicine and Health, University of Exeter, Exeter, United Kingdom

**Keywords:** intolerance of uncertainty, emotion, negative, positive, risk, ambiguity

## Abstract

Individuals high in self-reported Intolerance of Uncertainty (IU) tend to view uncertainty as unbearable and stressful. Notably, IU is transdiagnostic, and high levels of IU are observed across many different emotional disorders (e.g., anxiety, depression). Research has primarily focused on how IU evokes and modulates emotional states such as fear and anxiety. However, recent research suggests that IU may have relevance for a broader range of emotional states. Here, an online survey was conducted to examine whether IU evokes and modulates a range of negative (e.g., fear/anxiety, sadness/upset, anger/frustration, disgust) and positive (e.g., happiness/joy, excitement/enthusiasm, surprise/interest) emotional states. Findings within a community sample (*n* = 231) revealed that individuals with higher levels of IU report: (1) that uncertainty in general and uncertainty under ambiguity are more likely to evoke negative emotional states and less likely to evoke positive emotional states, (2) that uncertainty under risk is less likely to evoke positive emotional states, and (3) that uncertainty heightens existing negative emotional states and dampens existing positive emotional states. Importantly, these IU-related findings remained when controlling for current experiences of general distress, anxious arousal, and anhedonic depression. Taken together, these findings suggest that IU is involved in evoking and modulating a wide array of emotional phenomena, which likely has relevance for transdiagnostic models and treatment plans for emotional disorders.

## Introduction

Encountering uncertainty is common in everyday life (e.g., awaiting the result of a job interview or medical test). Both animals and humans attempt to reduce uncertainty, in order to optimally estimate the occurrence of motivationally relevant events (e.g., avoidance of threat, achieving reward, or safety) (1, 2). Current theoretical models and a wealth of empirical research suggests that uncertainty is aversive in and of itself, and thus engages the behavioral inhibition system (3) responsible for negative emotional states such as fear and anxiety (4–7). Alongside this literature, however, there is also an emerging body of research suggesting that uncertainty may bring about a psychological state that evokes and modulates other negative and positive emotional states as well (8–14). For instance, in a recent online survey study, Morriss et al. (13) found that different parameters of uncertainty (e.g., in general, in relation to negative and positive outcomes, under risk and ambiguity) evoked a range of negative and positive emotional states. Furthermore, Morriss et al. (13) observed uncertainty to heighten the intensity of negative emotional states (e.g., fear/anxiety, sadness/upset and frustration/anger), and dampen the intensity of positive (e.g., happiness/joy and, excitement/enthusiasm), emotional states, respectively.

Despite progress in understanding how uncertainty and emotion intersect (11, 13, 15–17), very little is understood as to how individual differences contribute to uncertainty and emotion interactions (15, 18). A growing body of research has started to highlight how the transdiagnostic risk factor, Intolerance of Uncertainty (IU), the tendency to view uncertainty as unbearable and stressful (19, 20), is associated with greater experience of negative emotional states such as fear and anxiety (21–23). Importantly, new evidence suggests that IU may be involved in evoking and modulating other negative and positive emotional states as well (24–30). For instance, higher levels of IU are associated with greater expression of negative emotions such as anger in individuals reporting more generalized anxiety disorder symptoms (25), as well as those with obsessive compulsive checking (30). Furthermore, higher levels of IU are associated with dampening of positive emotions in individuals reporting more generalized anxiety disorder symptoms (27), reduced reward anticipation in individuals with depression (28), and greater appraisals of threat for uncertain situations with a potential positive outcome (29). Such findings suggest that even when faced with uncertainty in positive contexts, individuals with high IU may actively dampen positive emotional experiences or have difficulty maintaining positive emotional experiences (e.g., blunted feelings of excitement and joy). Interestingly, populations with generalized anxiety disorder, social anxiety disorder, and obsessive compulsive disorder have also reported difficulties in experiencing positive affect or savoring positive affect (31–33), thus it is possible that high IU may be a pathway to dampened positive emotions in these populations.

IU has been identified as a transdiagnostic risk factor for emotional disorders such as anxiety, depression, obsessive-compulsive, post-traumatic stress, and eating disorders (34). Notably, a handful of promising results from recent clinical studies demonstrate that IU scores are reduced through transdiagnostic (35), general (36), and IU specific (37) cognitive-behavioral therapy protocols for emotional disorders such as anxiety and depression (e.g., primarily for anxiety). However, without further empirical research as to how IU is linked to emotional experiences more broadly, it is difficult to fully realize the potential of targeting IU in transdiagnostic treatments for singular and co-occurring emotional disorders (38, 39). For instance, if IU-based beliefs evoke and modulate a range of emotional states, then targeting IU-based beliefs may lead to treatment gains in regulating emotional responses across a range of situations (e.g., tolerating negative emotions and savoring positive emotions under uncertainty) that may be relevant to a number of different emotional disorders.

Prior research on uncertainty, emotion, and IU interactions has focused on a narrow range of parameters of uncertainty and emotional states (e.g., singular parameters and states) in isolation. To provide a more comprehensive picture of how uncertainty, emotion and IU interactions operate, in the following study, we conducted an online survey to examine whether IU is uniquely involved in evoking and modulating a range of negative (e.g., anxiety/fear, anger/frustration, sadness/upset, disgust) and positive (e.g., excitement/enthusiasm, surprise/interest, and happiness/joy) emotional states under different parameters of uncertainty (e.g., in general, in relation to negative and positive outcomes, under risk, and under ambiguity). Based on prior research highlighting that higher IU is associated with the tendency to experience greater negative affect [for review see Carleton (5)], and to some extent lesser positive affect [or reward sensitivity (28)] under uncertainty, we hypothesized that:

(1) Higher levels of IU would be significantly associated with greater elicitation of negative emotions and lesser elicitation of positive emotions in response to different parameters of uncertainty.
(2) Higher levels of IU would be significantly associated with heightened intensity of existing negative emotional states, and reduced intensity of existing positive emotional states when encountering uncertainty.

Similar to prior research on IU (40–42), we assessed the specificity of self-reported IU (19) on evoking and modulating emotional states under uncertainty by controlling for current experiences of general distress, anxious arousal, and anhedonic depression, measured *via* the Mini Mood and Anxiety Questionnaire (43).

## Materials and methods

The research presented here is a secondary analysis of an existing dataset by Morriss et al. (13).

### Participants

A total of 231 participants completed the online survey (see Table 1). Participants were recruited through advertisements across various social media platforms (Facebook, Twitter, and Instagram). To take part, participants had to be equal to or above 18 years old. There were no exclusion criteria. All participants provided virtual informed consent prior to their participation in the online survey. The study procedure was approved by the University of Reading Research Ethics Committee.

**TABLE 1:** Demographic characteristics of the participants.

Demographic variables	*N*	Median (standard deviation; range) or %
Age*	218	24 (12.47; 18−76)
**Gender**		
Female	172	74.46%
Male	47	20.35%
Other	8	3.46%
Unknown/not specified	4	1.73%
**Sexual orientation**		
Heterosexual	167	72.29%
LGBTQ+	43	18.61%
Unknown/not specified	21	9.09%
**Ethnicity**		
White	152	65.80%
Asian	25	10.82%
Black/African/Caribbean	15	6.49%
Latinx	12	5.19%
Middle eastern	5	2.16%
Multi-ethnic	4	1.73%
Other	1	0.43%
Unknown/not specified	17	7.36%
**Nationality**		
European	105	45.45%
North American	85	36.80%
Asian	16	6.93%
South American	5	2.16%
African	3	1.30%
Australasian	2	0.87%
Unknown/not specified	15	6.49%

Standard deviations are indicated in parenthesis. *13 cases noted as NA in the data were removed when calculating statistics for age.

### Materials

#### Uncertainty and emotion questionnaire

A novel questionnaire was previously developed to examine the interplay between uncertainty and a range of emotional experiences (41). The questions relevant to the current study are summarized in further detail below [for all questions see the Supplementary material of Morriss et al. (13)].

#### Uncertainty as an elicitor of emotional states

Five questions examined the reported frequency of discrete negative and positive emotions in relation to five distinct parameters of uncertainty [e.g., general, valenced outcomes (negative, positive), risk, and ambiguity]. Participants could select one or more of the following emotion categories in response to each of the five questions: happiness/joyful, sadness/upset, fearful/anxious, disgusted, angry/frustrated, surprised/interested, excited/enthusiastic, and confused.

The first question asked participants to indicate the emotions they commonly associated with “uncertainty generally.” The following two questions specifically focused on uncertainty in relation to the valence of the potential outcomes, with one question asking participants to indicate the emotions they commonly associated with “uncertainty in relation to potentially negative outcomes (i.e., exam situations, job applications)” and the other referring to “uncertainty in relation to potentially positive outcomes (i.e., exam situations, job applications).” The final two questions asked participants to select the emotions they commonly associated with uncertainty in relation to risk and ambiguity, respectively. The question related to risk was phrased as “uncertainty when you can predict the possible outcomes” with the example “i.e., in a job application, you know that you will either be successful or unsuccessful.” The question related to ambiguity was phrased as “uncertainty when you can’t predict the possible outcomes because there are many potential outcomes” with the example “i.e., your employer is considering merging departments, potentially resulting in a change of contract type, new role, promotion, or redundancy.”

#### Uncertainty as a modulator of existing emotional states

Six questions examined the modulatory impact of uncertainty on the experience of six existing emotional states. The six discrete emotion categories were: happy/joyful, sad/upset, fearful/anxious, disgusted, angry/frustrated, and excited/enthusiastic. Participants were asked to indicate the extent to which encountering uncertainty would impact the intensity of an existing emotional state on a 5-point Likert scale (1 = weaker, 5 = stronger). Example item: “*If you were feeling happy/joyful, would encountering uncertainty in your day to day life make this emotional state*….”

For this study, the Cronbach’s Alpha for the positive questions combined (happy/joyful and excited/enthusiastic) was α = 0.73 and for the negative questions combined (sad/upset, fearful/anxious, disgusted, and angry/frustrated) was α = 0.87.

### The intolerance of uncertainty scale-12

The Intolerance of Uncertainty Scale-12 measures reactions toward encountering uncertainty, situations that are ambiguous and future events (19). A total of 12 items are scored on a 5-point Likert Scale where 1 = “Not at all characteristic of me” and 5 = “Entirely characteristic of me,” with the higher scores indicating higher levels of intolerance of uncertainty. An example item would be: “Unforeseen events upset me greatly.” Cronbach’s alpha for all questions on the IUS-12 was α = 0.91.

### Mini mood and anxiety questionnaire

The Mini mood and anxiety questionnaire (Mini-MASQ) is used to assess current symptoms of depression and anxiety, and contains a list of possible feelings, sensations, and experiences (43). It consists of 26 items (Example item: “Felt really happy”) scored on a 5-point scale from 1 = “Not at all” to 5 = “Extremely,” where users indicate which number best describes their experience of the past week. Cronbach’s alpha for the total score on the Mini-MASQ was α = 0.92 and on the sub-scales; general distress was α = 0.92 (8 items), anxious arousal was α = 0.88 (10 items), and anhedonic depression was α = 0.85 (8 items).

### Procedure

Participants responded to an online advertisement *via* various social media platforms (i.e., Facebook, Twitter, and Instagram) and followed a secure link that led them to the online survey hosted on Jisc Online Survey.^1^ Following a brief description of the study, participants provided informed consent and then a series of demographic questions, including: date of birth, gender identity, sexual orientation, ethnicity, and nationality. This was followed by the completion of 18 questions that comprised the Uncertainty and Emotion questionnaire [only questions relating to how uncertainty elicits and modulates emotions are reported here, for all questions see the Supplementary material of Morriss et al. (13)]. Lastly, participants completed the IUS-12 and Mini-MASQ self-report questionnaires. The order of the questionnaires was the same for all participants. The survey took approximately 20 min to complete.

### Data reduction

Self-report data from the Uncertainty and Emotion questionnaire were averaged across negative categories and positive categories separately (for the results from the individual emotions, please see the Supplementary material).

#### Uncertainty as an elicitor of emotional states

For each question, self-reported responses to the eight emotion categories were coded as “1” for experience of the specific emotion and “0” for no experience of the specific emotion. The values (1’s and 0’s) for each question were then summed separately based on two emotion categories: “Negative Emotions” (Fearful/Anxious, Confused, Angry/Frustrated, Sadness/Upset, and Disgusted) and “Positive Emotions” (consisting of Excited/Enthusiastic, Happiness/Joyful, and Surprised/Interested).

#### Uncertainty as a modulator of existing emotional states

For each question, self-reported responses consisted of a value from 1 to 5. Self-report data were averaged across two emotion categories: “Negative Emotions” (Fearful/Anxious, Angry/Frustrated, Sadness/Upset, and Disgusted) and “Positive Emotions” (consisting of Excited/Enthusiastic and Happiness/Joyful).

### Data analyses

Statistical analyses were performed using RStudio version 4.1.1 (RStudio, PBC). The psych package was used to compute Cronbach’s alpha on scales and sub-scales. The ppcor package was used for correlation and partial correlation analyses. Plots and figures were formulated using ggpubr, Hsmic, and reshape2.

Due to the ordinal nature of the Uncertainty and Emotion questionnaire, non-parametric Spearman Rank Correlational tests were used to determine the strength and direction of the relationship between IU and negative and positive emotional states. To ensure that any significant relationships between IU and the negative and positive emotional states were specific to IU and not generally related to tendencies to experience anxiety and depression symptoms, partial non-parametric Spearman Rank Correlations were conducted between IU and negative and positive emotional states, while controlling for the total and sub-scale scores on the Mini-MASQ separately.

## Results

### IUS and mini-MASQ

The average scores for the IUS, combined total and three sub-factors of the Mini-MASQ are displayed in Table 2 (histograms of the IUS and Mini-MASQ scores can be found in the Supplementary material).

**TABLE 2:** Descriptive statistics (mean, standard deviation, median, and total possible range) for the IUS-12 and Mini-MASQ.

	Mean	SD	Median	Possible range
IUS-12	34.74	10.68	35.00	12−60
**Mini-MASQ sub-factors**				
General distress	20.43	8.50	20.00	8−40
Anxious arousal	17.33	7.63	15.00	10−50
Anhedonic depression	24.35	6.91	25.00	8−40
Mini-MASQ total	62.11	18.31	61.00	26−130

Higher scores on the IUS were significantly associated with higher scores on the Mini-MASQ [*r*(229) = 0.46, *p* < 0.01].

### Intolerance of uncertainty as an elicitor of emotions during different parameters of uncertainty

The average frequency for self-reported positive emotions and negative emotions across five uncertainty parameters are displayed in Table 3.

**TABLE 3:** Descriptive statistics (mean, standard deviation, median, and possible range) for the frequency of summated negative and positive emotions within the five parameters of uncertainty.

	Mean	SD	Median	Possible range
**General uncertainty**				
Negative emotions	2.19	1.22	2	0−5
Positive emotions	0.47	0.71	0	0−3
**Potentially negative outcome**				
Negative emotions	2.22	1.17	2	0−5
Positive emotions	0.12	0.36	0	0−3
**Potentially positive outcome**				
Negative emotions	0.58	0.78	0	0−5
Positive emotions	1.68	0.94	2	0−3
**Risk**				
Negative emotions	0.88	0.85	1	0−5
Positive emotions	1.04	0.90	1	0−3
**Ambiguity**				
Negative emotions	2.03	1.26	2	0−5
Positive emotions	0.40	0.66	0	0−3

#### Emotions associated with general uncertainty

**FIGURE 1:**
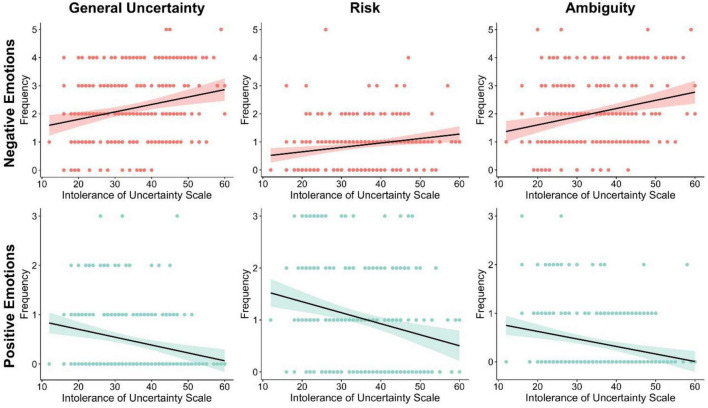
Higher IU scores are associated with: (1) greater elicitation of negative emotional states and lesser elicitation of positive states under general uncertainty and ambiguity, and (2) lesser elicitation of positive emotional states under risk.

As expected, under general uncertainty, higher scores on the IUS were associated with higher frequencies of negative emotions, [*r*(229) = 0.21, *p* < 0.01; see Figure 1]. The relationship between IUS and frequency of negative emotions remained significant after controlling for Mini-MASQ scores [*r*(228) = 0.15, *p* < 0.05], anxious arousal [*r*(228) = 0.15, *p* < 0.05], and anhedonic depression [*r*(228) = 0.20, *p* < 0.01], but not general distress [*r*(228) = 0.12, *p* = 0.06]. Furthermore, in situations with general uncertainty, higher scores on the IUS were associated with lower frequencies of positive emotions, [*r*(229) = −0.26, *p* < 0.01; see Figure 1]. This effect continued to be significant after controlling for scores on the Mini-MASQ [*r*(228) = −0.21, *p* < 0.01], general distress [*r*(228) = −0.20, *p* < 0.01], anxious arousal [*r*(228) = −0.25, *p* < 0.01], and anhedonic depression [*r*(228) = −0.23, *p* < 0.01].

#### Emotions associated with uncertain situations with potential negative outcomes

In uncertain situations with a potential negative outcome, IUS was not significantly associated with the frequency of negative [*r*(229) = 0.10, *p* = 0.12] or positive emotions [*r*(229) = 0.002, *p* = 0.98].

#### Emotions associated with uncertain situations with potential positive outcomes

For uncertain situations with a potential positive outcome, higher scores on the IUS were significantly associated with higher frequencies of negative emotions [*r*(229) = 0.15, *p* < 0.05]. However, this effect became non-significant when controlling for Mini-MASQ [*r*(228) = 0.05, *p* = 0.41], general distress [*r*(228) = 0.03, *p* = 0.68], anxious arousal [*r*(228) = 0.08, *p* = 0.24], and anhedonic depression [*r*(228) = 0.13, *p* = 0.055]. In addition, for uncertain situations with a potential positive outcome, no significant relationship between IUS and the frequency of positive emotions was observed [*r*(229) = −0.07, *p* = 0.26].

#### Emotions associated with risk

During situations with risk, higher scores on the IUS were significantly associated with higher frequencies of negative emotions [*r*(229) = 0.25, *p* < 0.01; see Figure 1]. This remained significant after controlling for anxious arousal [*r*(228) = 0.18, *p* < *0.01*] and anhedonic depression [*r*(228) = 0.22, *p* < *0.01*]. However, when controlling for total Mini-MASQ scores [*r*(228) = 0.13, *p* = 0.053] and general distress [*r*(228) = 0.11, *p* = 0.08] the relationship between IUS and the frequency of negative emotions became non-significant.

For situations with risk, higher scores on the IUS were significantly associated with lower frequencies of positive emotions [*r*(229) = −0.25, *p* < 0.01; see Figure 1]. This remained significant after controlling for the Mini-MASQ [*r*(228) = −0.16, *p* < 0.05], general distress [*r*(228) = −0.16, *p* < 0.05], anxious arousal [*r*(228) = −0.23, *p* < 0.01], anhedonic depression [*r*(228) = −0.20, *p* < 0.01].

#### Emotions associated with ambiguity

As expected, during ambiguity, higher scores on the IUS were associated with a higher frequency of negative emotions [*r*(229) = 0.24, *p* < 0.01; see Figure 1]. The relationship between IUS and the frequency of negative emotions remained significant after controlling for Mini-MASQ [*r*(228) = 0.21, *p* < 0.01], general distress [*r*(228) = 0.19, *p* < 0.01], anxious arousal [*r*(228) = 0.22, *p* < 0.01], and anhedonic depression [*r*(228) = 0.23, *p* < 0.01].

During ambiguity, higher scores on the IUS were associated with smaller frequencies of positive emotions [*r*(229) = −0.24, *p* < 0.01; see Figure 1]. This relationship remained significant after controlling for the Mini-MASQ [*r*(228) = −0.22, *p* < 0.01], general distress [*r*(228) = −0.22, *p* < 0.01], anxious arousal [*r*(228) = −0.25, *p* < 0.01], and anhedonic depression [*r*(228) = −0.22, *p* < 0.01].

### Intolerance of uncertainty as a modulator of emotions

**FIGURE 2:**
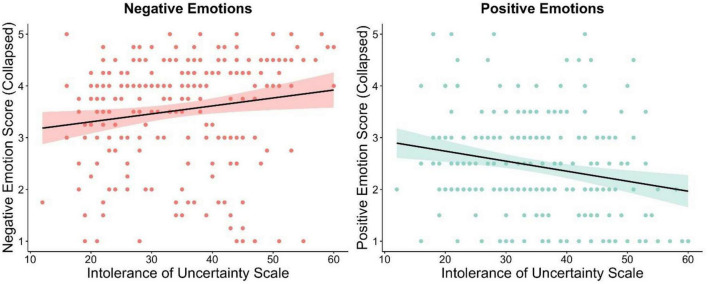
Higher IU scores are associated with increasing the intensity of existing negative emotions and decreasing the intensity of existing positive emotions when encountering uncertainty.

The collapsed average of the negative and positive emotions are displayed separately in Table 4. As predicted, higher scores on the IUS were significantly associated with increasing the intensity of existing negative emotions when encountering uncertainty, [*r*(229) = 0.22, *p* < 0.01; see Figure 2]. Notably, the effect of IUS on negative emotions remained after controlling for the total Mini-MASQ score [*r*(228) = 0.14, *p* < 0.05], and the Mini-MASQ subscales of anxious arousal [*r*(228) = 0.18, *p* < 0.01] and anhedonic depression [*r*(228) = 0.18, *p* < 0.01]. A similar pattern was also observed for the general distress subscale but it was not statistically significant [*r*(228) = 0.13, *p* = 0.06].

**TABLE 4:** Descriptive statistics (mean, standard deviation, median, and possible range) for the intensity of negative and positive emotions.

	Mean	SD	Median	Possible range
Negative emotional states	3.53	1.03	3.75	1−5
Positive emotional states	2.45	0.96	2.50	1−5

Furthermore, as expected higher scores on the IUS were significantly associated with decreasing the intensity of existing positive emotions when encountering uncertainty, [*r*(229) = −0.22, *p* < 0.01; see Figure 2]. As above, the effect of IUS on positive emotions remained after controlling for the total Mini-MASQ score [*r*(228) = −0.21, *p* < 0.01], and the Mini-MASQ subscales of general distress [*r*(228) = −0.20, *p* < 0.01], anxious arousal [*r*(228) = −0.22, *p* < 0.01], and anhedonic depression [*r*(228) = −0.21 *p* < 0.01].

## Discussion

The key findings from this study revealed that individuals with high levels of IU report: (1) that uncertainty in general and uncertainty under ambiguity are more likely to evoke negative emotional states and less likely to evoke positive emotional states, (2) that uncertainty under risk is less likely to evoke positive emotional states, and (3) that uncertainty heightens existing negative emotional states and dampens existing positive emotional states. Crucially, these IU-related findings emerged, regardless of current experiences of general distress, anxious arousal, and anhedonic depression. In sum, IU evokes and modulates a wide array of emotional phenomena. Such findings will likely inform current transdiagnostic models and therapies for emotional disorders.

IU-related differences in evoking negative (e.g., anger, anxiety, sadness, but not disgust) and positive (e.g., joy, excitement, surprise) emotional states primarily emerged for uncertainty in general, uncertainty under ambiguity, and to some extent uncertainty under risk, but not uncertainty in relation to potential negative and positive outcomes. Arguably, IU-related differences in evoking negative and positive emotional states may have been observed for uncertainty in general and uncertainty under ambiguity because these parameters involve a higher level of uncertainty (e.g., more unknowns) and are therefore may be inherently more salient and aversive (13, 22). Surprisingly, IU-related differences were not observed to impact the experience of negative and positive emotions for uncertainty in relation to potential negative and positive outcomes, although the results were in the anticipated direction but not significant (e.g., higher IU was associated with more negative emotions and less positive emotions). These findings are at odds with past research demonstrating a role of IU in modulating psychophysiological and neural markers during conditions with uncertain negative and positive outcomes (22, 23). The lack of IU-related differences in influencing emotional experiences for uncertainty in relation to potential negative and positive outcomes in this study may be due to several different factors. Firstly, for scenarios with uncertainty and clear valenced outcomes that are highly motivationally relevant (e.g., job interviews, exams) there may less variation in self-reported responses. Secondly, in this study, the crude form of measurement used (e.g., total frequency of emotional states), rather than, or in relation to, the intensity of emotional states (44–46) may have reduced variation in self-reported responses.

In the current study, IU-related differences were found to modulate existing negative and positive emotions when encountering uncertainty in everyday life. For instance, individuals with higher IU reported that negative emotions (e.g., anger, anxiety, but not disgust) were heightened when encountering uncertainty, whereas positive emotions (e.g., joy, excitement) were dampened when encountering uncertainty. These findings are in line with new theoretical positions and evidence demonstrating that IU can modulate a range of negative and positive emotional experiences, beyond that of fear and anxiety (9, 10, 25, 27, 28, 30). Importantly within the present study, the majority of significant associations between IU and the evocation and modulation of emotional experiences remained significant, while controlling for current symptoms of general distress, anxious arousal, and anhedonic depression. Such findings suggest that IU may be a robust individual differences predictor of uncertainty and emotion interactions.

In line with current models of IU (4, 5, 7), the findings suggest that individuals with high IU are likely to experience negative emotions such as fear and anxiety under uncertainty. However, the findings here also support newer theoretical models such as the Uncertainty Distress Model (10), by demonstrating that individuals with high IU may also frequently experience other negative emotions, such as anger and sadness as well (15, 25, 30), and have difficulty experiencing or engaging with positive emotions under uncertainty (27, 28). Furthermore, these findings sit alongside past research that has observed heightened negative emotions and dampened positive emotions in populations with anxiety disorders (31–33). Given that IU is higher in such populations, it is possible that IU may be a potential pathway for the regulation of negative and positive emotions in different emotional disorder populations. A crucial next step is to identify whether individuals with high IU from different populations (e.g., different cultures, ages, and within clinical samples) label and appraise emotional experiences under uncertainty in a similar way or not. Addressing this research question will inform current transdiagnostic and disorder specific treatment plans for emotional disorders, where high levels of IU are common (38, 39).

The study had a few notable strengths and weaknesses. Firstly, the IU and the Mini-MASQ questionnaires show good validity and reliability across different populations in Europe, the Americas, and Asia (47–52). That being said, the sample here is primarily female, white, European, and English speaking, thus further replication in more diverse samples is required to fully assess the generalizability of the findings. Secondly, the online survey data were collected during the COVID-19 pandemic, thus the results from some individuals regarding intolerance of uncertainty and their emotional experience may be have been more extreme due to this context (e.g., may have been more affected by the COVID-19 pandemic due to current health or working conditions). Thirdly, the present study examined interactions between IU, different parameters of uncertainty (risk and ambiguity), and emotion, which is considerably rare in the English-speaking literature, where IU has been primarily examined under risk and ambiguity in the context of fear and anxiety (24, 29, 41, 53). Although, the questionnaire developed to examine different parameters of uncertainty here used narrow examples of risk and ambiguity in work and education scenarios (e.g., job interviews, exams). Therefore, future research would benefit from extending this investigation to other types of scenarios common to everyday life (e.g., relationships, leisure, play etc.).

In conclusion, the findings reported suggest that IU is involved in evoking and modulating a wide array of emotional phenomena. This line of research likely has relevance for understanding the role of intolerance of uncertainty as a transdiagnostic treatment target for emotional disorders.

## Data availability statement

The original contributions presented in this study are included in the article/Supplementary material, further inquiries can be directed to the corresponding author.

## Ethics statement

The studies involving human participants were reviewed and approved by the University of Reading. The patients/participants provided their written informed consent to participate in this study.

## Author contributions

JM, CH, and HD conceived the ideas for this research. JM developed the ideas for this research, designed the study, obtained funding, and collected the data. KG conducted the data reduction and statistical analyses. JM and KG wrote the original manuscript draft. CH and HD edited the manuscript and contributed to the interpretation. All authors approved the final manuscript.

## Footnotes

1
https://www.onlinesurveys.ac.uk/
