# Supplementary material for: Intolerance of uncertainty heightens negative emotional states and dampens positive emotional states
Source: Front Psychiatry. 2023 Mar 22;14:1147970. doi: 10.3389/fpsyt.2023.1147970 (PMC10073686; doi:10.3389/fpsyt.2023.1147970)
Supplement: Supplementary file 1 [file Data_Sheet_1.docx]

**Supplementary material for intolerance of uncertainty heightens negative emotional states and dampens positive emotional states**

**Figure 1**

**
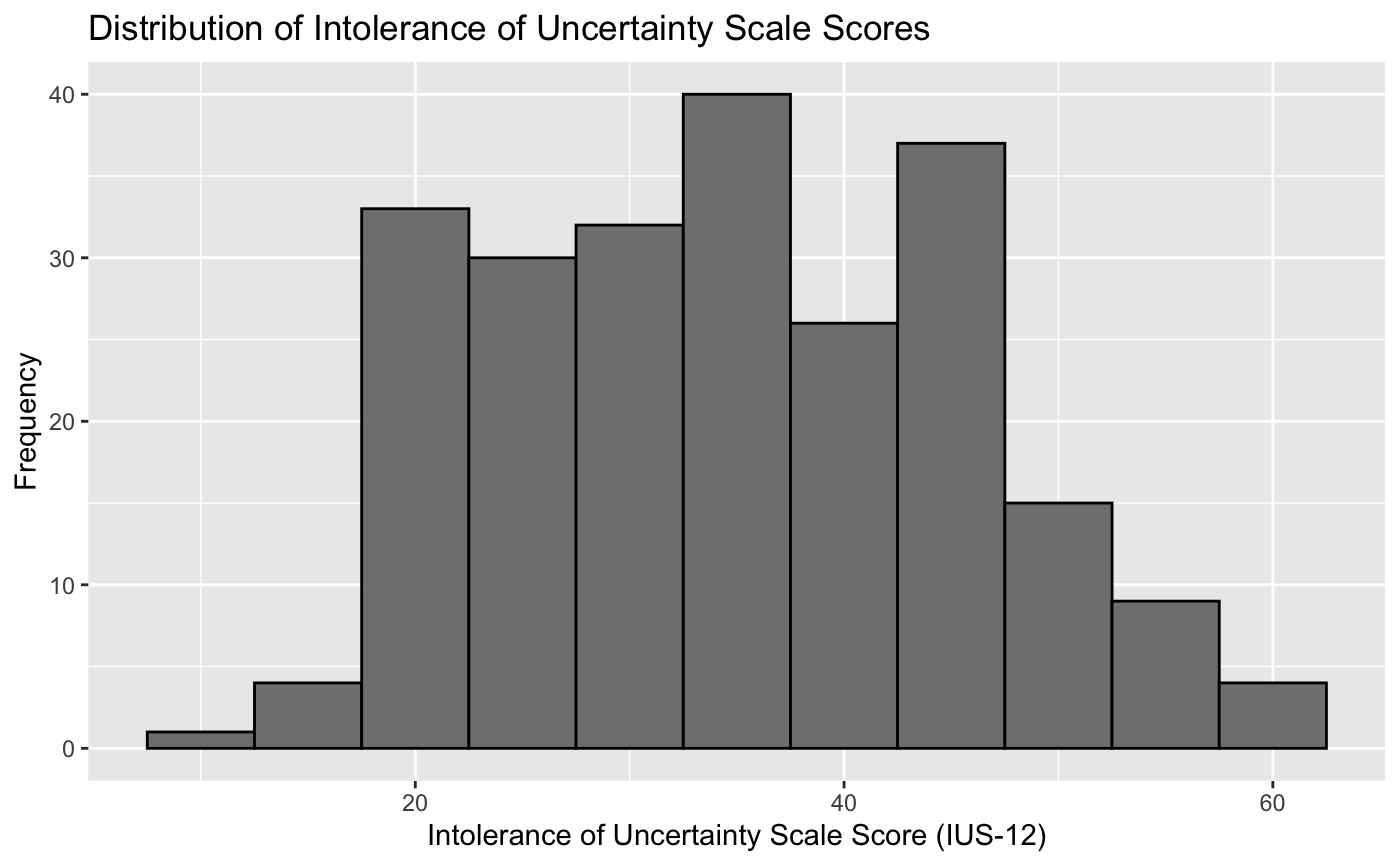
**

**
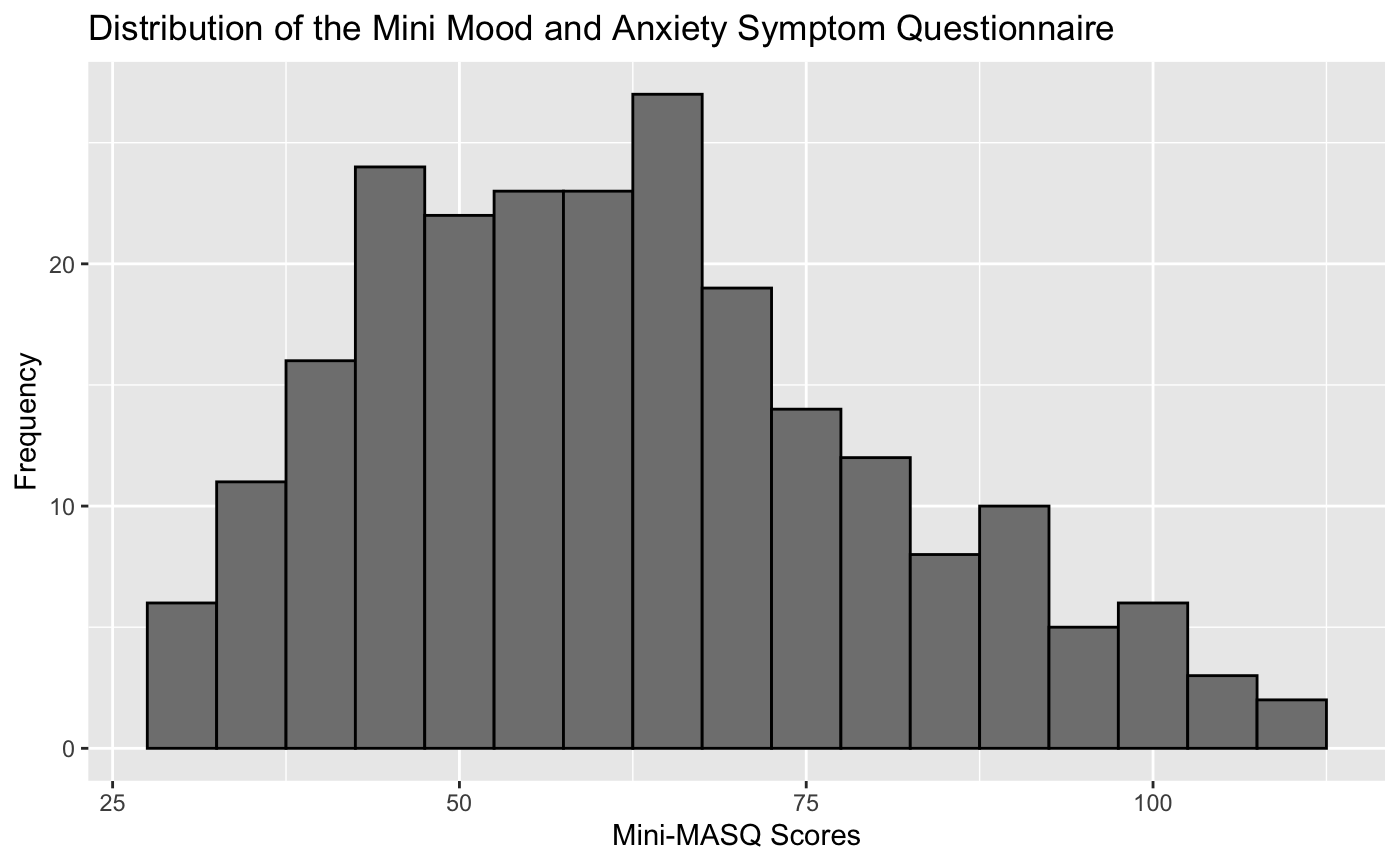
**

**Intolerance of Uncertainty as an elicitor of individual emotions**

**Figure 2**

*Correlation Matrix between IUS-12 and the frequency of Individual emotions under general uncertainty*

**
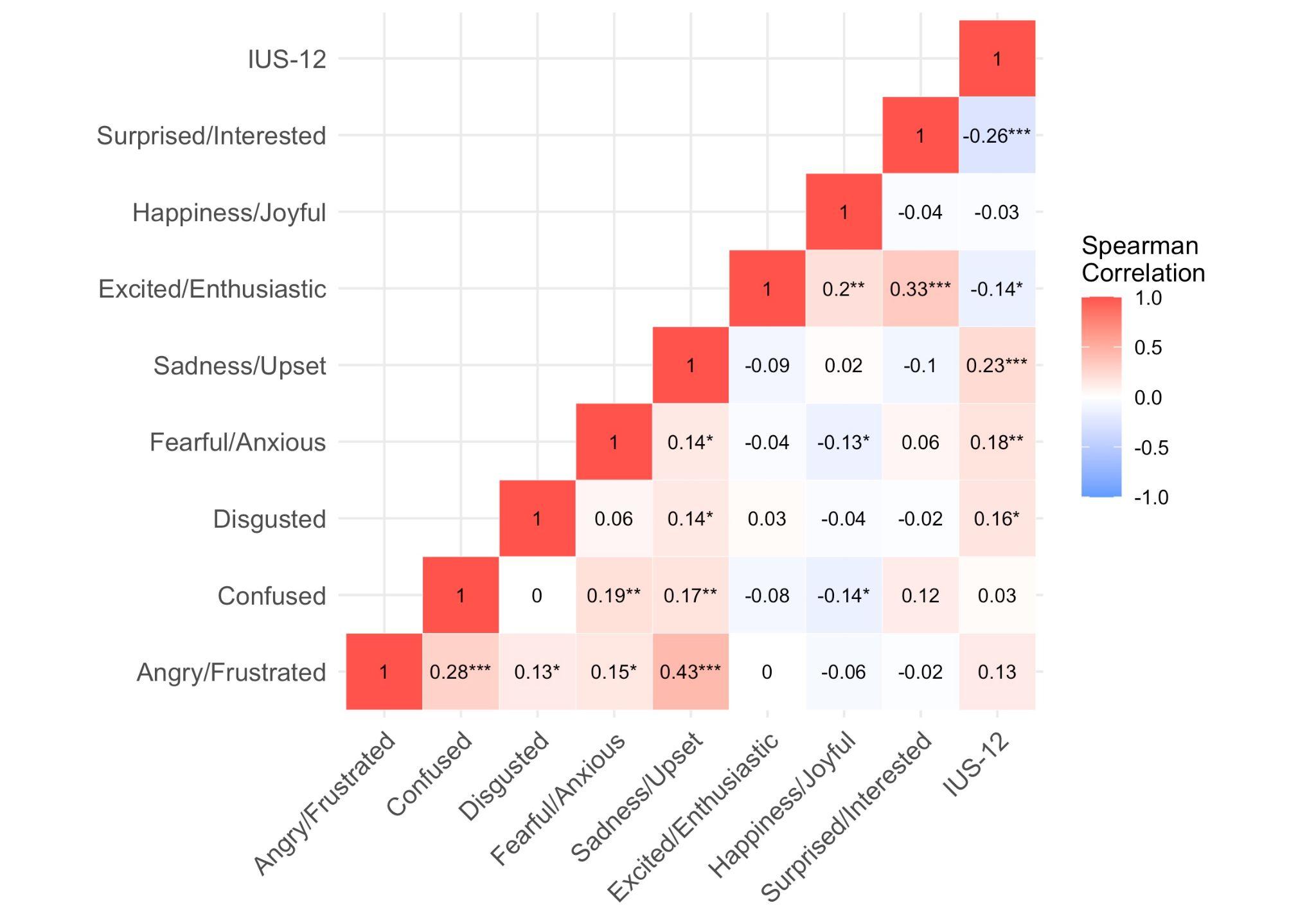
**

*Notes*.

* *p* < .05, ** *p* < .01 and *** p< .001

**Figure 3**

*Correlation Matrix between IUS-12 and the frequency of Individual emotions for uncertain situations with potentially negative outcomes*


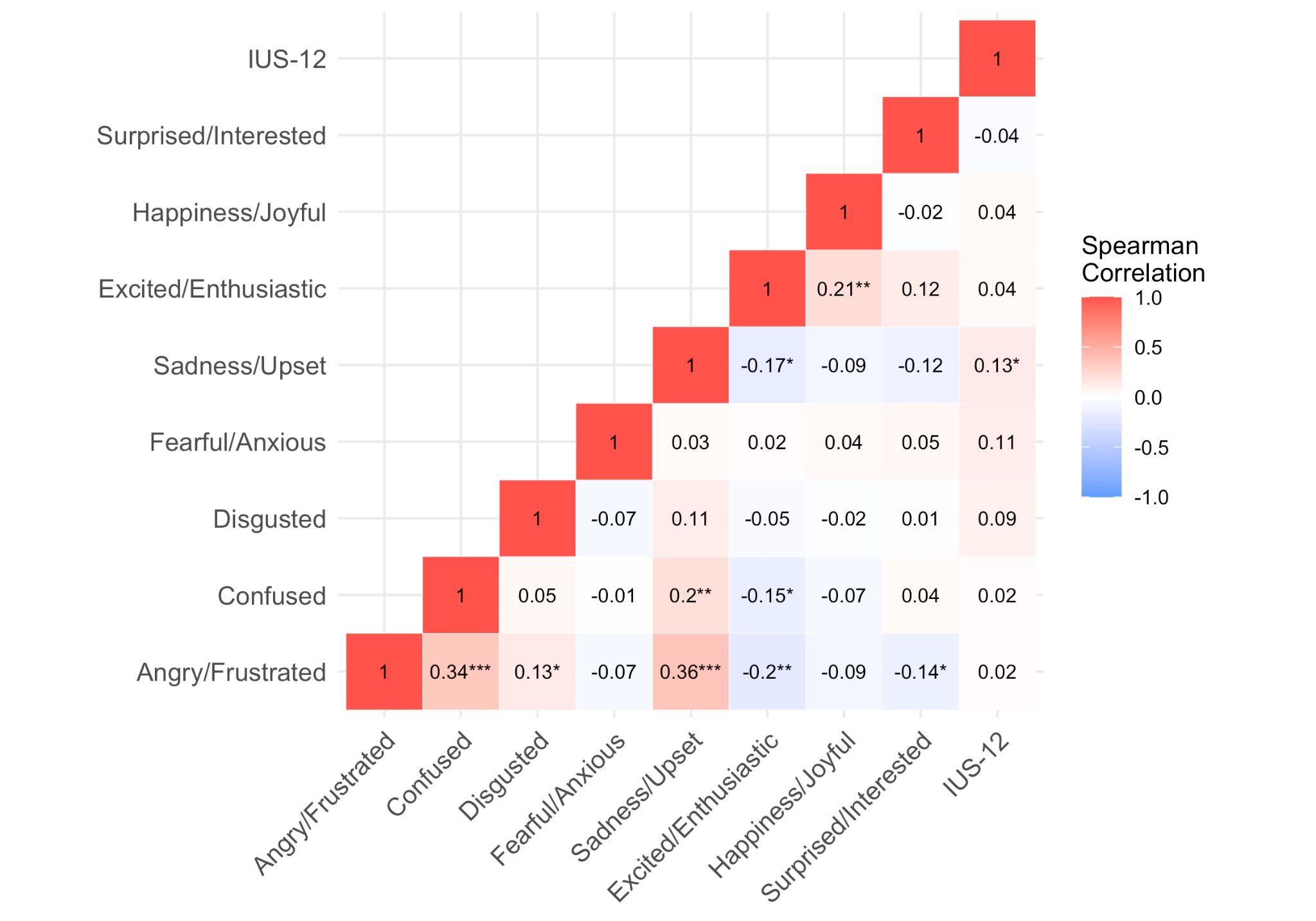


*Notes*.

* *p* < .05, ** *p* < .01 and *** p< .001

**Figure 4**

*Correlation Matrix between IUS-12 and the frequency of Individual emotions for uncertain situations with potentially positive outcomes*

**
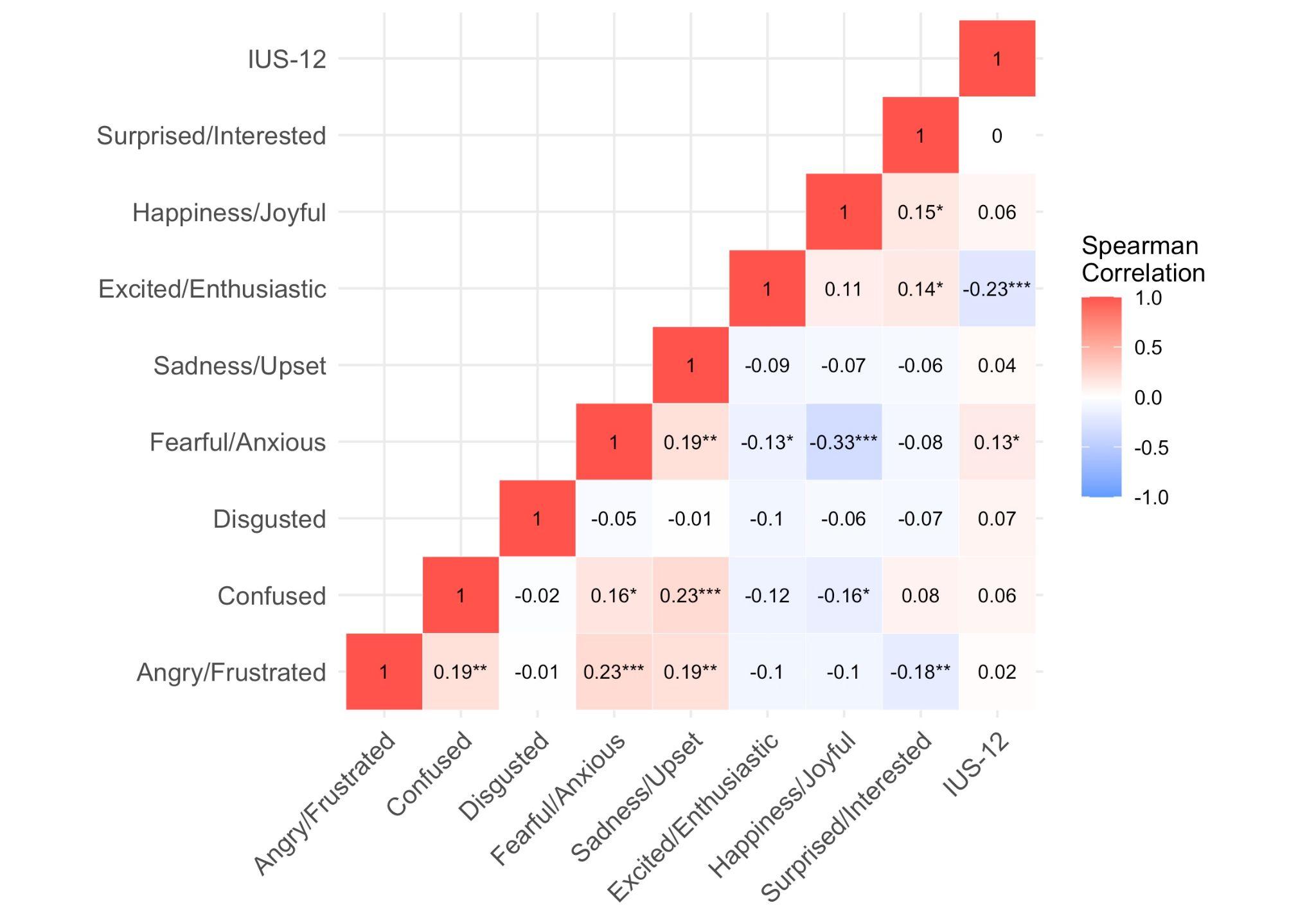
**

*Notes*.

* *p* < .05, ** *p* < .01 and *** p< .001

**Figure 5**

*Correlation Matrix between IUS-12 and the frequency of Individual emotions under risk*

**
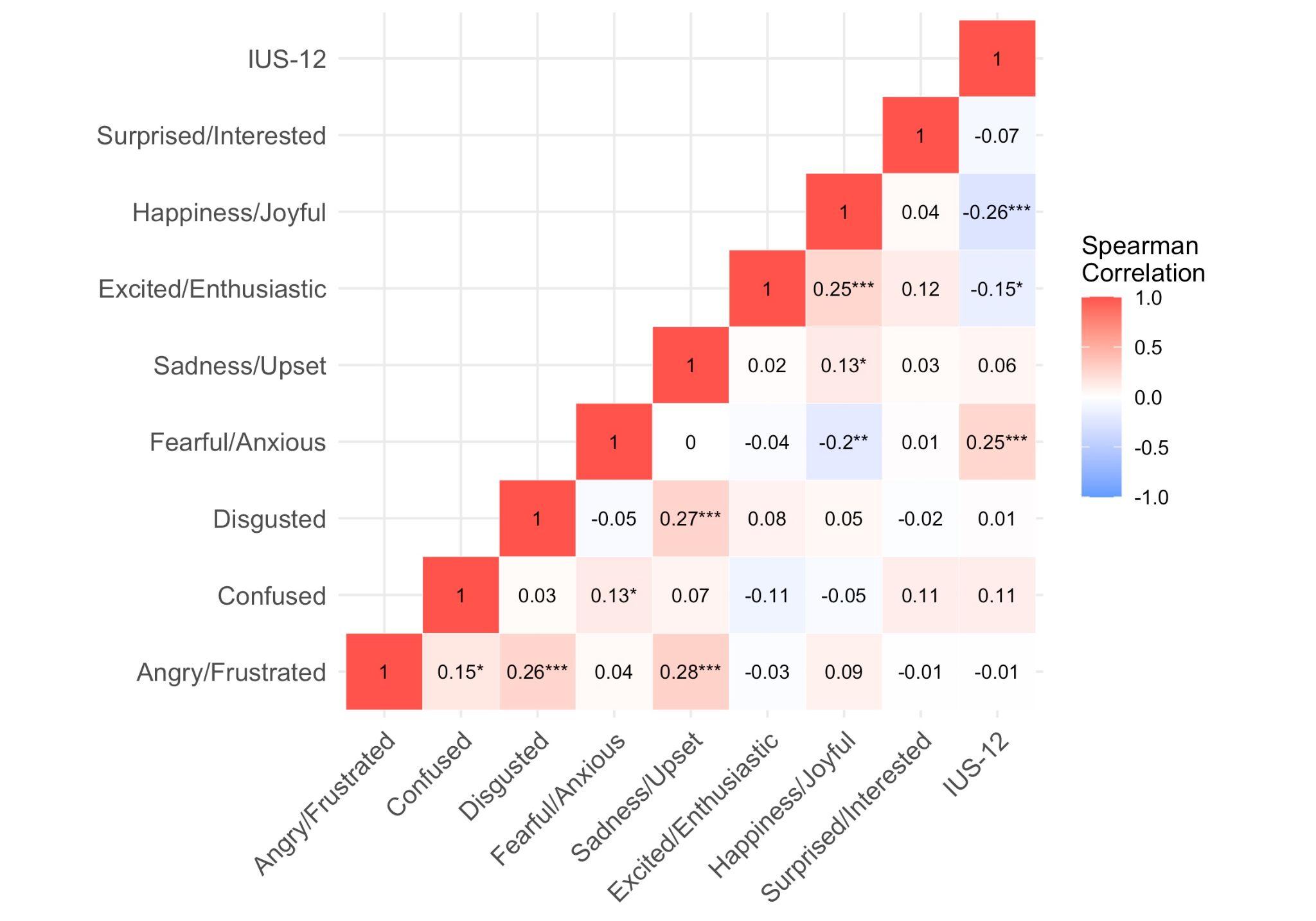
**

*Notes*.

* *p* < .05, ** *p* < .01 and *** p< .001

**Figure 6**

*Correlation Matrix between IUS-12 and the frequency of Individual emotions under ambiguity*

*
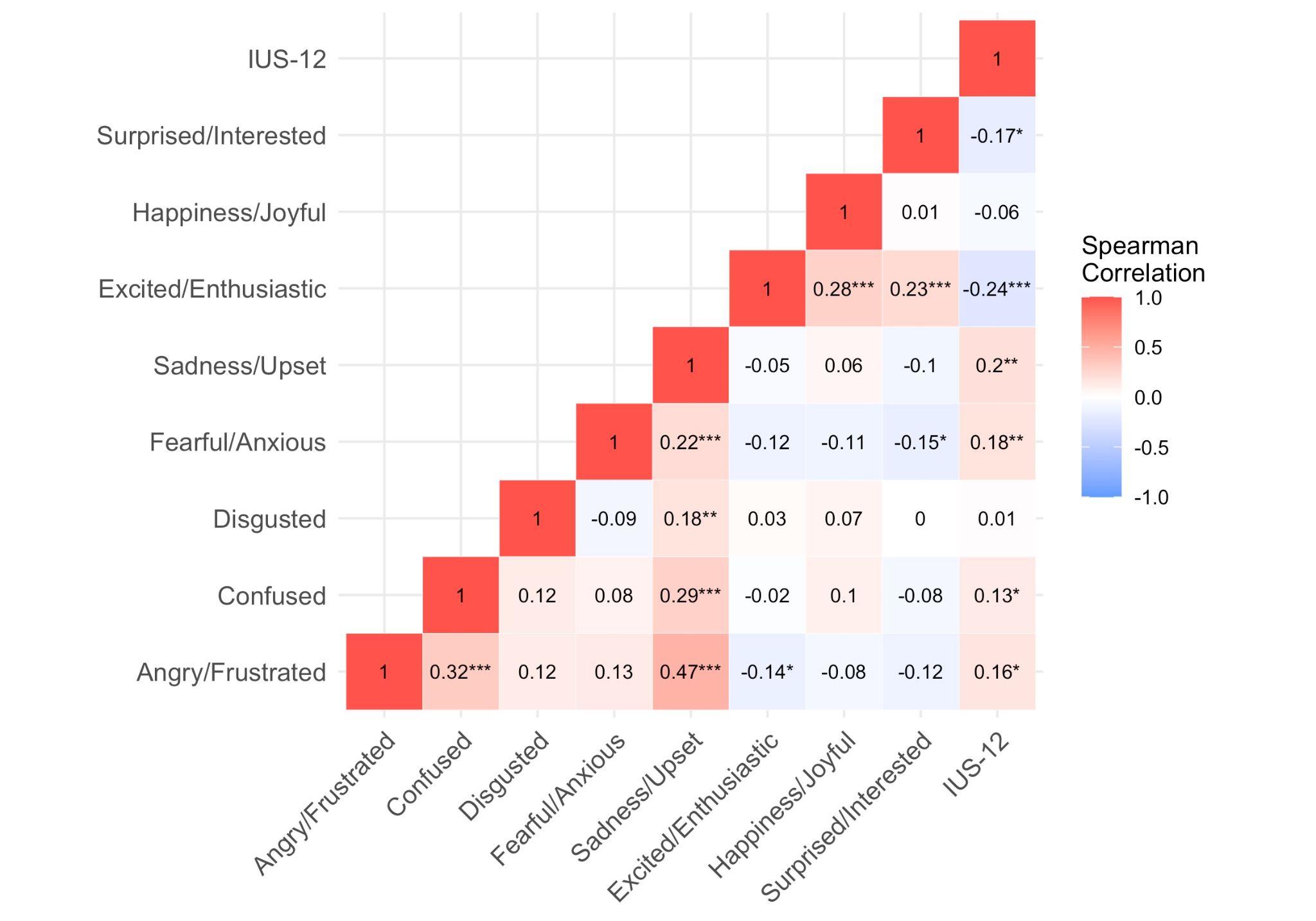
*

*Notes*.

* *p* < .05, ** *p* < .01 and *** p< .001

**Intolerance of Uncertainty as a modulator of emotions**

**Table 1**

*Descriptive statistics (Mean, SD, Median, Total possible range) of individual emotions*

|  | Mean | SD | Median | Total Possible Range |
| --- | --- | --- | --- | --- |
| Angry/Frustrated | 3.72 | 1.21 | 4.00 | 1-5 |
| Disgusted | 3.06 | 0.93 | 3.00 | 1-5 |
| Excited/Enthusiastic | 2.68 | 1.14 | 3.00 | 1-5 |
| Fearful/Anxious | 3.82 | 1.37 | 4.00 | 1-5 |
| Happiness/Joyful | 2.23 | 1.01 | 2.00 | 1-5 |
| Sadness/Upset | 3.53 | 1.27 | 4.00 | 1-5 |

**Figure 7**

*Spearman Rank Correlation matrix for individual emotions and Intolerance of Uncertainty*

**
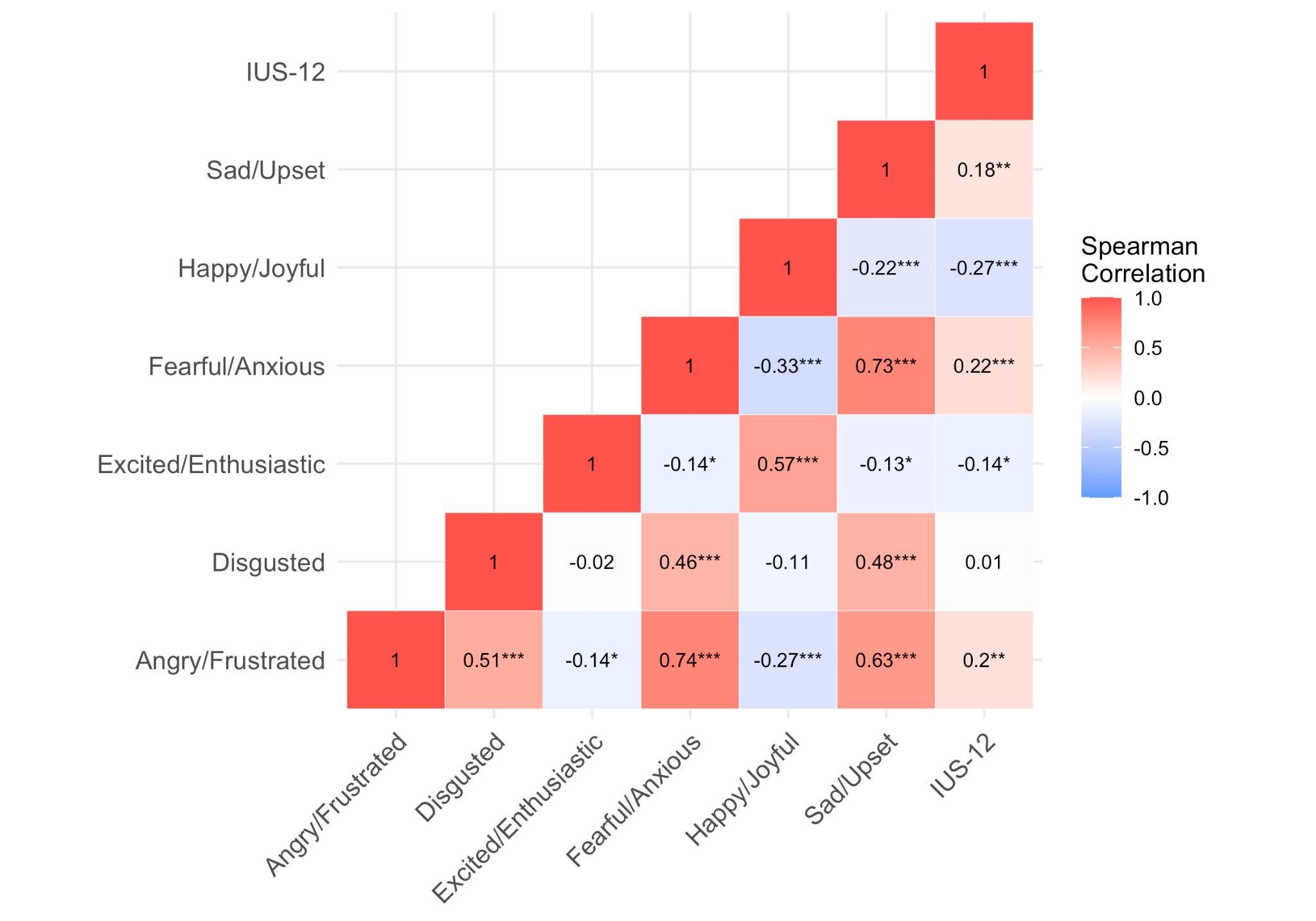
**

*Notes*.

* *p* < .05, ** *p* < .01 and *** p< .001

**Table 2**

*Spearman Rank Correlations between Negative Emotions with and without disgust, and the IUS-12 before and after controlling for Mini-MASQ and its subscales*

|  | Negative Emotions | Negative Emotions omitting Disgust |
| --- | --- | --- |
| Correlation with IUS-12 | r(229)=0.22, *p*< .01 | r(229)=0.25, *p*< .01 |
| After Controlling for |  |  |
| Mini-MASQ | r(228)=0.14, *p* <.05 | r(228)=0.17, *p*<.01 |
| General Distress | r(228)=0.13, *p*=.06 | r(228)=0.15, *p*<.05 |
| Anxious Arousal | r(228)=0.18, *p*<.01 | r(228)=0.21, *p*<.01 |
| Anhedonic Depression | r(228)=0.18, *p*<.01 | r(228)=0.21, *p*<.01 |

The average score for negative emotions (Fearful/Anxious, Angry/Frustrated and Sadness/Upset) was 3.69 (SD=1.17) out of a possible score of 5.
